# Endocrine polyautoimmunity: Mechanistic insights and the future of AI-driven diagnostics

**DOI:** 10.17179/excli2025-8748

**Published:** 2025-11-05

**Authors:** Shabnam Heydarzadeh, Raziyeh Abooshahab, Maryam Zarkesh, Mehdi Hedayati

**Affiliations:** 1 Cellular and Molecular Endocrine Research Center, Research Institute for Endocrine Molecular Biology, Research Institute for Endocrine Sciences, Shahid Beheshti University of Medical Sciences, Tehran, Iran; 2 Curtin Medical School, Curtin University, Bentley, Australia

**Keywords:** polyautoimmunity, autoimmune thyroid diseases, autoimmune polyendocrine syndrome, autoantibodies, pathology, artificial intelligence

## Abstract

The most prevalent form of polyautoimmunity is autoimmune thyroid diseases (AITD), which frequently coexist with other autoimmune disorders and often act as a central conductor in the symphony of autoimmunity. Due to overlapping clinical manifestations, diagnosing polyautoimmunity presents significant clinical challenges. Patients with AITD exhibit increased susceptibility to additional autoimmune disorders, in which the exact etiology and underlying mechanisms of these associations remain incompletely understood. In this review, we aim to discuss how mechanistic insights contribute to our understanding of the associations between endocrine autoimmune diseases to recognize shared immunological, genetical, and pathological patterns for these diseases. Recent findings, including epitope spreading, cytokine imbalance, shared thyroidal and non-thyroidal autoantibodies, and common genetic susceptibilities, are highlighted. Additionally, the integration of artificial intelligence (AI) into autoimmune diagnostics is addressed, underscoring AI's potential to enhance early detection, improve diagnostic accuracy, and support personalized treatment approaches. By recognizing distinct immunological, genetical and pathological patterns within polyautoimmunity, clinicians and researchers can more effectively target the root causes of immune dysregulation, enabling improved management through personalized strategies and advanced AI-driven tools.

See also the graphical abstract(Fig. 1).

## Introduction

Polymorbidity in autoimmune diseases is quite frequent, with as many as 25 % of patients diagnosed with one autoimmune disorder also developing another at some point in their lives (Mohan and Ramesh, 2003[72]). Among such overlapping disorders, autoimmune thyroid diseases (AITDs), including Hashimoto's thyroiditis and Graves' disease, are particularly prominent (Ai et al., 2003[1]). AITD is one of the most prevalent autoimmune conditions and frequently coexists with other autoimmune diseases in the same individual (Cárdenas Roldán et al., 2012[21]). Indeed, studies indicate that AITD often serves as a central component in polyautoimmunity, the presence of multiple autoimmune diseases in a single patient (Rojas-Villarraga et al., 2012[86]). This tendency for co-occurrence is not a mere coincidence but is believed to arise from shared etiological factors, including common genetic susceptibilities, immune system dysregulation, and environmental triggers (Rojas-Villarraga et al., 2012[86], Franco et al., 2013[40]). Recognizing AITD as a potential initiator or chaperone in the development of additional autoimmune disorders underscores the need for an integrated approach to these patients.

One well-recognized category of polyautoimmune disorders is the autoimmune polyendocrine syndromes (APS) (Kahaly and Frommer, 2018[51]). These syndromes are defined by the coexistence of at least two autoimmune endocrinopathies, often accompanied by autoimmune involvement of non-endocrine organs. AITD is a frequent component of APS, especially in APS type II (Schmidt's syndrome), which classically involves autoimmune Addison's disease (primary adrenal insufficiency) in combination with AITD and/or type 1 diabetes mellitus (T1DM) (Betterle et al., 2002[12], Majeroni and Patel, 2007[64]). In fact, AITD is present in approximately 70-80 % of patients with APS type II (Majeroni and Patel, 2007[64]). Other autoimmune conditions, such as vitiligo or chronic atrophic gastritis leading to pernicious anemia, may also co-occur in a subset of APS patients (Majeroni and Patel, 2007[64]). This multifaceted relationship between thyroid autoimmunity and other organ-specific disorders means that the diagnosis of AITD should prompt vigilant screening for additional autoimmune diseases characteristic of APS (Li et al., 2020[58]). Conversely, patients presenting with disorders like Addison's disease or T1DM benefit from careful monitoring for emerging thyroid dysfunction (Meling Stokland et al., 2022[70]). Early recognition of these connections allows for more comprehensive and proactive management of individuals at risk for multiple autoimmune conditions.

Despite the well-documented associations between AITD and other autoimmune diseases, the mechanistic links underlying their coexistence remain incompletely understood. Our study seeks to bridge this gap, offering new insights and directions for future researches. The overlapping clinical manifestations of different autoimmune disorders can also pose significant diagnostic challenges, as symptoms may mimic or obscure one another (Reynolds and Putterman, 2023[85]). This underscores the need for deeper insight into how and why these diseases cluster. To address this gap, the present review examined the immunological, genetic, and pathological intersections between AITD and coexisting autoimmune conditions. This review assessed the prevalence and impact of thyroid-specific autoantibodies**,** notably anti-thyroid peroxidase (anti-TPO), anti-thyroglobulin (anti-Tg), and TSH receptor antibodies in patients with multiple autoimmune diseases. These autoantibodies, hallmark features of AITD, can sometimes exert effects beyond the thyroid gland, and their presence in other autoimmune disorders may offer clues to shared disease mechanisms. Clarifying these common threads can help determine whether a unifying immunopathological link underlies the coexistence of AITD with other disorders. Finally, this review considers the emerging role of artificial intelligence (AI) in improving the diagnosis and management of polyautoimmune conditions. By integrating AI-based analytical tools with clinical and immunological insights, it may become possible to recognize polyautoimmunity more efficiently and tailor personalized treatment strategies for affected patients. 

## Main Actors in the Co-Existence of AITD with Other Autoimmune Diseases

**Figure 2:**
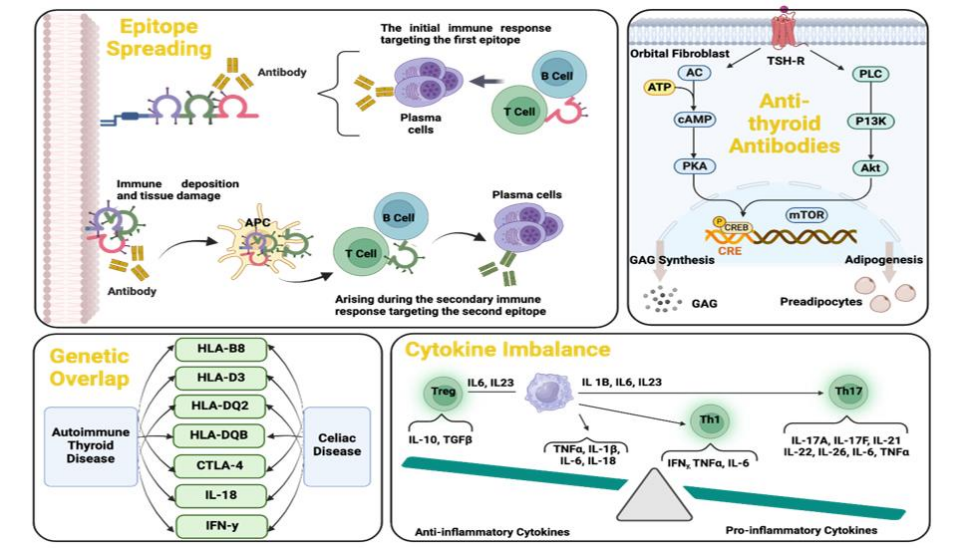
The main actors in the co-existence of autoimmune diseases. This figure illustrates the underlying factors that contribute to autoimmune disorders, including epitope spreading, anti-thyroid antibodies, genetic overlap, and cytokine imbalance.

**Figure 3:**
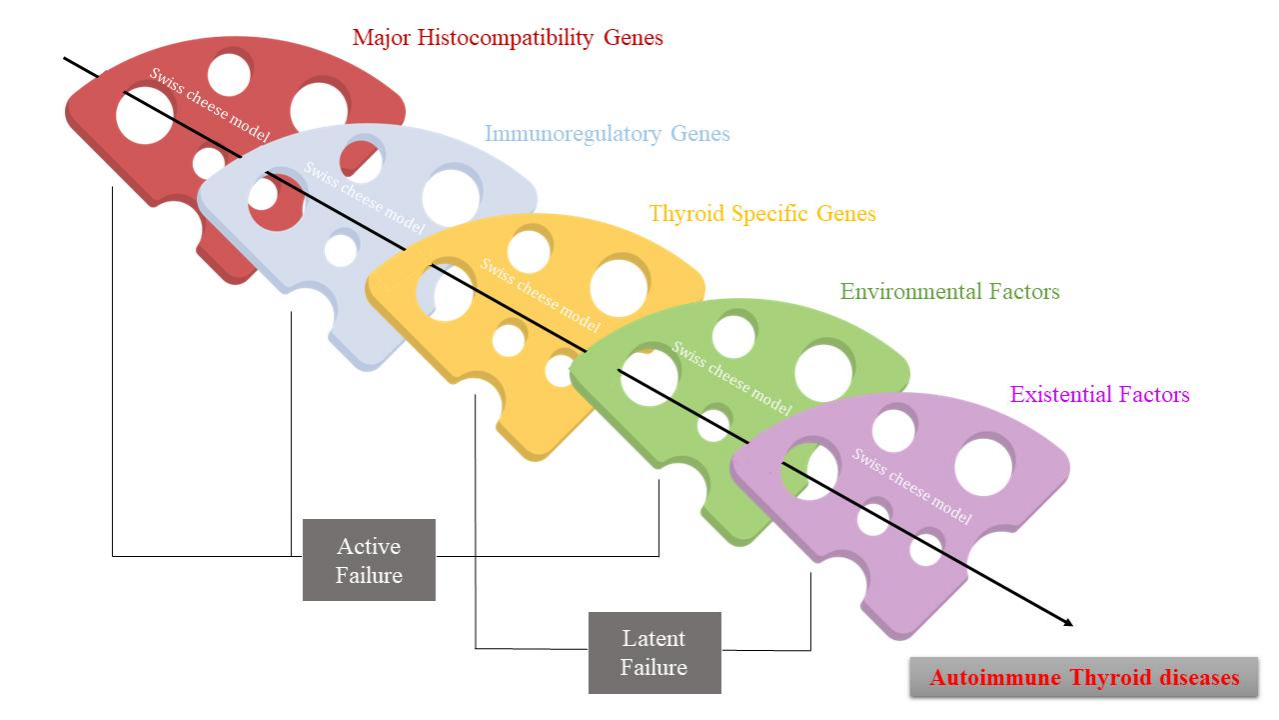
Etiology of thyroid autoimmunity. The development of autoimmune thyroid disease is a result of multiple events, which are described in a model named the “Swiss cheese model”. A Swiss cheese model for the causation of autoimmune thyroid disease, showing the effect of cumulative weaknesses lining up to allow a catastrophic event to occur, like the holes in slices of cheese.

Multiple lines of evidence indicate that when AITD co-occurs with other autoimmune disorders, this is not a random coincidence but the result of shared pathogenic mechanisms. The autoimmune tautology hypothesis posits that different autoimmune diseases have common roots (Cojocaru et al., 2010[29]). Key factors contributing to the coexistence of AITD with additional autoimmune diseases include genetic susceptibilities, immune system dysregulation (such as cytokine imbalances and regulatory T cell dysfunction), epitope spreading, antigenic cross-reactivity, autoantibodies, and environmental triggers (Rydzewska et al., 2018[88], Liu et al., 2023[62]) (Figure 2(Fig. 2)). These factors act in concert, reminiscent of a Swiss cheese model in which multiple predisposing holes line up to precipitate overt polyautoimmunity (Figure 3(Fig. 3)).

### Inherent susceptibility and overlapping genes

**Figure 4:**
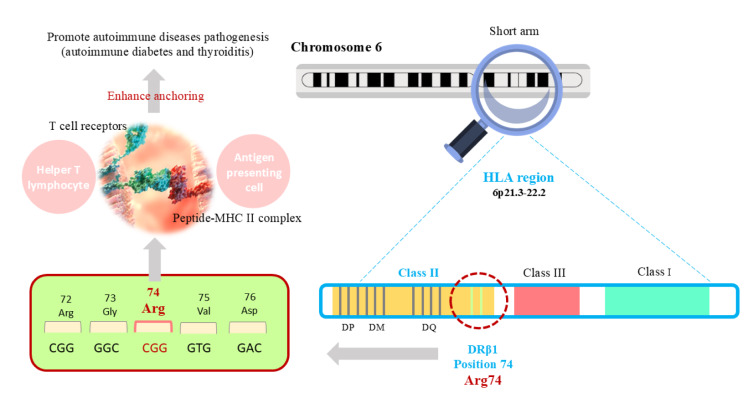
Amino acid signature within the HLA-DR peptide-binding pocket confers susceptibility to both autoimmune diabetes and thyroiditis. Arg-74 (Arg located in position 74 of the DRβ1 chain) plays a key role in anchoring T-cell receptors to the peptide-MHC II complex, contributing to the pathogenesis of multiple autoimmune diseases.

Hereditary factors play a central role in predisposing individuals to multiple autoimmune diseases (Ramos et al., 2015[83]). Family and population studies have long shown that AITD and other autoimmune disorders tend to cluster in families, pointing to inherited risk alleles (Brix and Hegedüs, 2012[19]). The most significant genetic contributors are variants in the HLA region, which influence antigen presentation and T-cell activation (Jacobson et al., 2008[48], Vargas-Uricoechea, 2023[104]). Specific HLA class II haplotypes are strongly associated with both AITD and other autoimmune diseases (Simmonds and Gough, 2004[92]). HLA-DR3 and HLA-DR4, along with their associated DQ variants (DQ2 and DQ8), are classic susceptibility alleles found at high frequency in Hashimoto's thyroiditis, Graves' disease, type 1 diabetes, and autoimmune Addison's disease (Simmonds and Gough, 2004[92], Dittmar et al., 2008[33], Tomer and Menconi, 2009[100]). A patient carrying the HLA-DR3-DQ2 or HLA-DR4-DQ8 haplotypes has an increased risk for developing both thyroid autoimmunity and type 1 diabetes, illustrating a genetic link between the two conditions (Tomer and Menconi, 2009[100]). Moreover, a distinct molecular signature in the HLA-DR pocket, defined by specific amino acids, significantly increases the risk of T1D and AITD. Arginine at position 74 of the DRβ1 chain may promote disease by enhancing interactions between the HLA-DR-peptide complex and the T-cell receptor. Thus, Arg-74 likely plays a key role in anchoring T-cell receptors to the peptide-MHC II complex, contributing to the pathogenesis of multiple autoimmune diseases (Menconi et al., 2010[71]) (Figure 4(Fig. 4)). Specific HLA alleles, including HLA-DRB1*03 and HLA-DRB1*04, have also been implicated in the co-occurrence of AITD with autoimmune gastritis (Zantut-Wittmann et al., 2004[118], Sartoris et al., 2024[89]), suggesting a common immunogenetic susceptibility in thyrogastric syndrome. 

Beyond HLA, numerous non-HLA genes that modulate immune function have been identified as shared susceptibility genes across multiple autoimmune diseases. Polymorphisms in genes encoding immunoregulatory proteins, such as cytotoxic T-lymphocyte antigen-4 (CTLA-4 ) and protein tyrosine phosphatase, non-receptor type 22 (PTPN22), are notable examples (Chistiakov and Turakulov, 2003[25], Thomas and Veerabathiran, 2025[98]). The CTLA-4 gene, which is crucial for downregulating immune responses, harbors variants that confer risk for both autoimmune thyroiditis and type 1 diabetes (Ikegami et al., 2006[47]), confirming it as a common susceptibility locus in families affected by both diseases. Likewise, the PTPN22 R620W polymorphism (rs2476601), which affects T-cell receptor signaling, is a well-known risk factor for type 1 diabetes and has been associated with AITD in many populations (Wu et al., 2005[112], Bottini et al., 2006[16]). These shared genetic risk factors partially explain why the two diseases frequently coexist. Other gene polymorphisms, interleukin-2 receptor alpha (IL2RA), forkhead box P3 (FOXP3), thyroglobulin (TG), CD25, CD40, CLEC16A, and FCRL3, among others, have been found to contribute to both thyroid autoimmunity and type 1 diabetes (Frommer and Kahaly, 2021[42]). Variations in IL2RA, which encodes the alpha chain of the IL-2 receptor essential for Treg cell activity, are associated with vulnerability to both diseases (Borysewicz-Sańczyk et al., 2020[14]). Additionally, variants of the FOXP3 gene, which influence regulatory T cells, have been associated with autoimmune responses in both the thyroid and pancreatic islets (Li et al., 2020[58]). It is worth noting that a single patient's unique genetic makeup might determine which combination of diseases they develop. For example, a person carrying HLA-DR3 and CTLA-4 variants might develop Hashimoto's thyroiditis and type 1 diabetes, whereas another with HLA-DR3 plus additional variants in AIRE or FEZF2 genes could develop Hashimoto's thyroiditis and autoimmune gastritis, since AIRE mutations predispose to a broad spectrum of autoimmunity as seen in APS type I (Peterson and Peltonen, 2005[77], Lee et al., 2023[55]). Overall, the common genetic denominators across AITD and other autoimmune diseases provide a fundamental explanation for their co-occurrence. 

### Immune dysregulation: loss of tolerance and cytokine imbalance 

**Figure 5:**
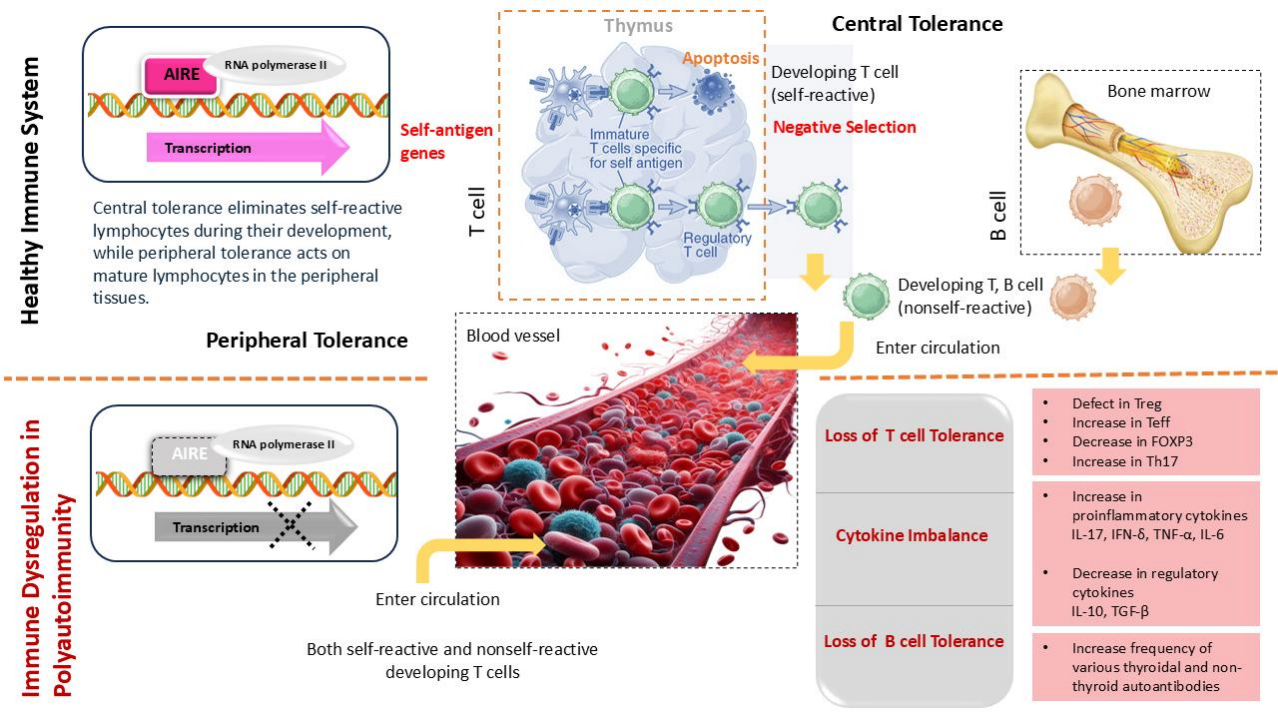
The difference between a healthy immune system and a dysregulated immune condition in polyautoimmunity. In the presence of the AIRE gene, self-reactive T cells of the thymus are deleted in a negative selection process, and only non-self-reactive developing T and B cells enter circulation (central tolerance). In the absence of the AIRE gene, both self-reactive and non-self-reactive developing T cells enter the circulation (loss of central tolerance and cytokine imbalance).

In addition to genetic predisposition, immune system dysregulation is a unifying feature in patients with multiple autoimmune diseases. A healthy immune system maintains tolerance to self-antigens through multiple mechanisms, including central tolerance, which is the deletion of self-reactive T cells in the thymus, a process partly governed by the AIRE gene and peripheral tolerance, which is the suppression of auto-reactive cells by regulatory T cells and immune checkpoints like CTLA-4 (Proekt et al., 2017[79], Li et al., 2020[58]). In polyautoimmunity, these tolerogenic mechanisms often fail broadly, not just for one organ. Evidence of widespread loss of tolerance can be seen in patients with AITD and concomitant autoimmune diseases. Individuals with coexisting AITD and type 1 diabetes exhibited defects in the number or function of regulatory T cells (Tregs), along with increased effector T cell activity, when compared to healthy controls (Bennett et al., 2001[9], Fontenot and Rudensky, 2005[39]) (Figure 5(Fig. 5)). A decrease in FOXP3-expressing Tregs alongside an increase in pro-inflammatory T helper 17 (Th17) cells has been observed in both autoimmune thyroiditis and type 1 diabetes (Bossowski et al., 2013[15], Li et al., 2016[57]). Th17 cells, characterized by their interleukin-17 (IL-17) production, are now recognized as important drivers of autoimmunity. An abundance of Th17 cells and elevated IL-17 levels have been documented in the thyroid tissue of Hashimoto's patients (Wiersinga, 2018[111]), as well as in the gastric mucosa of patients with autoimmune gastritis (Cascetta et al., 2024[22]), and are believed to exacerbate local inflammation. Thus, a cytokine imbalance skewed toward pro-inflammatory cytokines, including IL-17, IFN-γ, TNF-α, and IL-6, while moving away from regulatory cytokines such as IL-10 and TGF-β, represents a common immune profile across various autoimmune diseases (Yasuda et al., 2019[116]). In AITD, thyroid-infiltrating T cells secrete interferon-gamma (IFN-γ) and tumor necrosis factor-alpha (TNF-α), which contribute to thyroid follicular cell destruction (Ganesh et al., 2011[44], Ferrari et al., 2023[38]). Patients with concomitant AITD and another autoimmune condition often exhibited higher systemic levels of these pro-inflammatory cytokines than patients with either disease alone (Ferrari et al., 2023[38], Yao et al., 2024[115]), suggesting an additive effect of multiple autoimmune processes on the immune milieu. Another aspect of immune dysregulation in polyautoimmunity is the activation of B cells and the production of autoantibodies against multiple targets (Rydzewska et al., 2018[88]). A breakdown of B-cell tolerance can lead to the generation of autoantibodies not only to thyroid antigens but also to antigens of other tissues in the same individual (Das et al., 2018[31]). Patients with Hashimoto's thyroiditis have an increased frequency of various non-thyroid autoantibodies such as anti-gastric parietal cell, anti-intrinsic factor, anti-adrenal cortex, anti-pancreatic islet cell, and antinuclear antibodies (Boutzios et al., 2022[17], Tripolino et al., 2024[101]). The simultaneous presence of these autoantibodies reflects a broad auto-reactive B cell repertoire. In one study, anti-parietal cell antibodies (APCAs) were found in a significant proportion of Hashimoto's thyroiditis patients, correlating with a higher prevalence of anti-thyroid peroxidase antibodies in those individuals (Twito et al., 2018[102]). This implies a generalized propensity for B cells to break tolerance and target multiple organs. The breadth of autoantibody profiles in polyautoimmune patients results from immune dysregulation and serves as a tool for early diagnosis. 

It is more evident that individuals predisposed to polyautoimmunity often exhibit an immune system that is biased towards autoimmunity in general rather than a single-organ autoimmunity. Elevated inflammatory cytokines, such as those of the Th1 and Th17 types, and impaired regulatory mechanisms create a fertile ground for multiple autoimmune diseases to co-occur. This justifies a holistic immunomodulatory approach to treatment in the future, rather than addressing each autoimmune disease in isolation. 

### Epitope spreading and antigenic cross-reactivity

**Figure 6:**
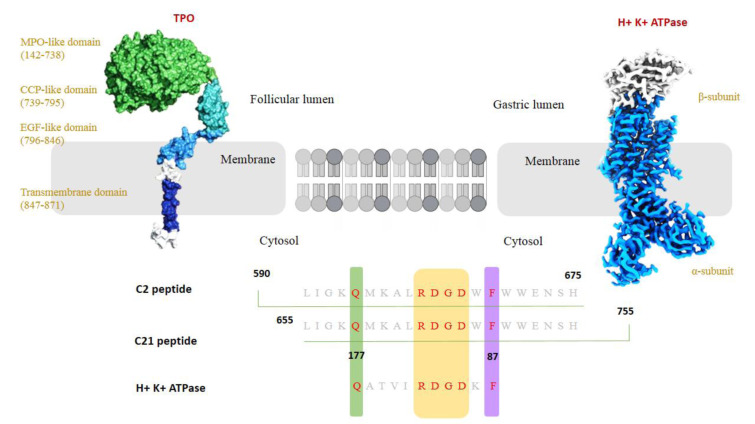
Amino acid sequence homology (A shared 11-residue peptide) between TPO of AITD and proton pump H^+^K^+^ ATPase antigen of gastric parietal cells.

A pivotal mechanism connecting one autoimmune disease to another is epitope spreading, the diversification of an immune response to new self-antigen epitopes over time (McLachlan and Rapoport, 2017[69]). Initially, an autoimmune process may be triggered by an immune response to a specific antigen in one organ. As tissue damage occurs, previously sequestered or cryptic antigens can be exposed, or inflammatory conditions may promote the presentation of additional self-epitopes (Benvenga and Guarneri, 2016[10]). The immune system, already in an activated state, may begin reacting to these new epitopes, which can belong to the same or a different organ. Over time, this cascade can result in multiple autoimmune diseases in the same patient (Benvenga and Guarneri, 2016[10], McLachlan and Rapoport, 2017[69]). In the context of AITD, epitope spreading may help explain why patients develop autoimmunity in other organs. A case in point is Hashimoto's thyroiditis, which destroys thyroid tissue, releasing thyroid antigens in abundance (Weetman, 2008[108]). One striking example is the thyrogastric syndrome, where patients have both autoimmune thyroiditis and autoimmune gastritis (Elisei et al., 1990[35]). Research suggests that TPO, a key thyroid antigen in Hashimoto's, shares a segment of amino acid sequence homology with the proton pump antigen of gastric parietal cells. A shared 11-residue peptide between TPO and the parietal cell H+/K+-ATPase has been identified, indicating a potential common epitope that could drive cross-reactive antibody responses (Elisei et al., 1990[35]) (Figure 6(Fig. 6)). In a patient with Hashimoto's, the ongoing anti-TPO immune response might inadvertently target the gastric mucosa if the immune system “sees” the parietal cell ATPase as containing an antigenic epitope similar to TPO (Boutzios et al., 2022[17]). Over time, this could lead to autoimmune gastritis. Therefore, antigenic cross-reactivity, a form of intermolecular epitope spreading, serves as a plausible link between the thyroid and gastric issues and autoimmunity (Boutzios et al., 2022[17]). 

Epitope spreading may occur in two ways: intramolecularly, where various epitopes of the same antigen trigger an immune response, and intermolecularly, where epitopes from different antigens could lead to autoimmunity affecting additional organs (Benvenga and Guarneri, 2016[10]). This process results in the appearance of various autoantibodies in patients with chronic autoimmune diseases. Patients with multiple autoimmune diseases often show a temporal sequence in their development; one disease (e.g., AITD) may precede another (e.g., pernicious anemia) by years (Bliddal et al., 2017[13], Zulfiqar and Andres, 2017[124]). During this interval, low-level autoantibodies to the second organ can sometimes be detected, hinting at epitope spreading before clinical disease (McLachlan and Rapoport, 2017[69]). An illustrative case is type 1 diabetes and AITD, in which many children with type 1 diabetes initially have only islet autoantibodies, but after several years, a subset begin to develop anti-thyroid antibodies, and then go on to manifest Hashimoto's hypothyroidism (Kordonouri et al., 2002[52], Jung et al., 2014[50], Herczeg et al., 2025[46]). This progression supports the idea that an active autoimmune condition like diabetes can lead to immune broadening and a second condition, including thyroiditis, via epitope spreading or by creating a pro-inflammatory environment (McLachlan and Rapoport, 2017[69]).

Molecular mimicry related to environmental triggers can be seen as a special case of epitope spreading where an exogenous antigen, such as a viral protein, shares epitopes with self-proteins (Cusick et al., 2012[30]). Chronic viral or bacterial infections might initiate an autoimmune response that later spreads to self-antigens in multiple organs (Münz et al., 2009[73]). Enteroviruses, which have long been implicated in type 1 diabetes pathogenesis, might also trigger autoimmunity in thyroid tissue through bystander damage or molecular mimicry (Weider et al., 2020[110], 2021[109]). 

### Anti-thyroid antibodies in thyroidal and extra-thyroidal diseases

**Figure 7:**
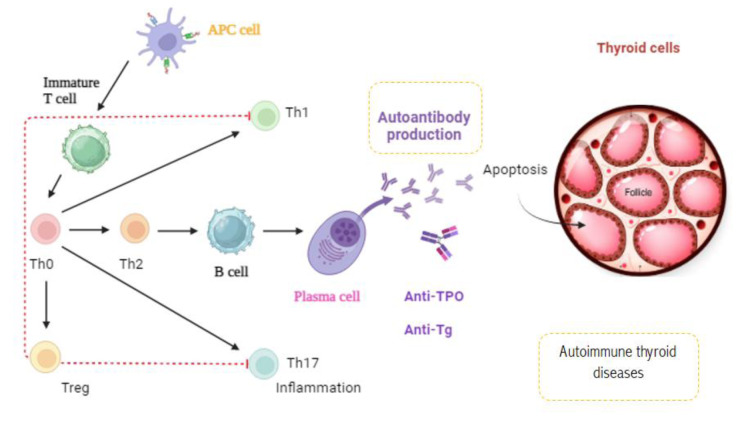
The mechanism of autoantibody production in thyroid autoimmune diseases. Autoantibody production stems from a breakdown of immune tolerance, where the body's immune system mistakenly attacks its own thyroid tissue. This involves generating antibodies against thyroid-specific antigens, such as Tg and TPO. The inflammatory response, apoptosis, and tissue damage lead to the characteristic symptoms and clinical manifestations of thyroid autoimmune diseases.

Thyroid autoantibodies are frequently found in individuals with AITD, as well as in those who show no signs of thyroid dysfunction. While the presence of anti-thyroid antibodies in other immune disorders is relatively common, individuals with AITD tend to develop antibodies against organs beyond the thyroid less commonly. In Figure 7(Fig. 7), we attempt to illustrate the mechanism of autoantibody production in thyroid autoimmune diseases. Long-term Hashimoto's patients may accumulate antibodies not only to thyroglobulin and TPO, but also to other thyroid or non-thyroid antigens (Silajdzija et al., 2022[91]). Subjects with dysregulated immune systems are more susceptible to the development of anti-thyroid antibodies. The frequency of anti-TPO antibodies in autoimmune thyroid disease is somewhat greater than that of anti-Tg antibodies. Unlike that, the presence of anti-TSHR antibodies in non-thyroid immunological illnesses is rarely documented (Fröhlich and Wahl, 2017[41]). It has been demonstrated that patients with GD and Graves' ophthalmopathy frequently exhibit a Th1 immune response in the early stages of these conditions. Antonelli and Benvenga (2018[4]) noted a shift from a Th1 to a Th2 profile in the inactive phase of GD and Graves' ophthalmopathy, indicating that both genetic and environmental factors contribute to the development of autoimmune phenomena in multiple organs, often characterized by a Th1 dominance during the active phases of these diseases (Antonelli and Benvenga, 2018[4]). Table 1(Tab. 1) (References in Table 1: Anaya et al., 2019[3]; Antonelli et al., 2007[5]; Atzeni et al., 2008[7]; Brenner et al., 2004[18]; Cárdenas Roldán et al., 2012[21]; Cellini et al., 2017[23]; Cindoruk et al., 2002[28]; Fattori et al., 2008[36]; Lahner et al., 2008[53]; Lopomo and Berrih-Aknin, 2017[63]; Malandrini et al., 2022[65]; Pyne and Isenberg, 2002[80]; Quidute et al., 2012[82]; Rashad et al., 2020[84]; Şimşek et al., 2020[93]; Souto Filho et al., 2020[95]; Tamer et al., 2016[97]; Vrijman et al., 2012[105]; Zhang et al., 2022[121]; Zhao et al., 2021[122]) shows the prevalence of thyroidal autoantibodies in other autoimmune diseases.

### Environmental triggers 

While genetics and immune dysregulation set the stage, environmental factors often act as triggers that can initiate or exacerbate multiple autoimmune diseases in a predisposed individual. These include infections, dietary components, drugs, and even physical or emotional stress (Rose, 2016[87]). In patients with AITD, specific environmental factors are recognized for triggering thyroid autoimmunity. For instance, high iodine intake may prompt Hashimoto's thyroiditis in those who are susceptible, while stress or infections can lead to Graves' disease (Brix and Hegedüs, 2012[19], Zimmermann and Boelaert, 2015[123]). Many of these same triggers can independently play a role in other autoimmune diseases.

Recent findings indicate that commensal microorganisms have a role in the pathogenesis of T1DM through the toll-like receptor signaling pathway, which is also implicated in AITD (Li et al., 2020[58]). Moreover, Helicobacter pylori in the stomach has been proposed to contribute to both autoimmune gastritis and even thyroid autoimmunity via generalized immune activation or molecular mimicry mechanisms (Wang et al., 2022[107]). 

The microbiome is an emerging factor, as the composition of gut microbiota influences systemic autoimmunity (Belkaid and Hand, 2014[8]). Dysbiosis might promote Th17 responses or impair Treg development, thereby fostering conditions such as type 1 diabetes and autoimmune thyroid disease concurrently (Su et al., 2020[96], Qin et al., 2025[81]). 

Vitamin D deficiency, partly influenced by diet and sunlight exposure, is another environmental factor linked to increased risk of both Hashimoto's thyroiditis and type 1 diabetes (Lebiedziński and Lisowska, 2023[54], Durá-Travé and Gallinas-Victoriano, 2024[34]). Prior research indicates that the Th-2 cell response significantly contributes to the pathogenesis of T1DM and AITD, while Vitamin D supplementation mitigates the aberrant activation of T cells and enhances immune homeostasis, implying a therapeutic potential for Vitamin D in coexisting autoimmune disorders (Gregori et al., 2002[45], Jeffery et al., 2015[49], Malik et al., 2016[66]).

Additionally, gender and hormonal factors play a significant role; females exhibit a greater tendency towards various autoimmune diseases. For instance, a woman diagnosed with one autoimmune condition, such as Hashimoto's, is at an increased risk for developing other diseases like lupus or rheumatoid arthritis, which may be linked to the hormonal modulation of the immune system (Desai and Brinton, 2019[32], Lin et al., 2023[60]). While sex hormones are not an environmental factor per se, life events like pregnancy, which can induce postpartum thyroiditis or unmask other autoimmune conditions, tie into this risk (Pearce, 2020[76]). All these factors, including infections, microbiome, nutrition, hormones, and aging, are thought to work in tandem with genetic and immunologic factors to determine whether a patient with AITD will remain with a single-organ disease or progress to a multi-autoimmune syndrome.

## Role of Epigenetics in the Pathogenesis of AITD

**Figure 8:**
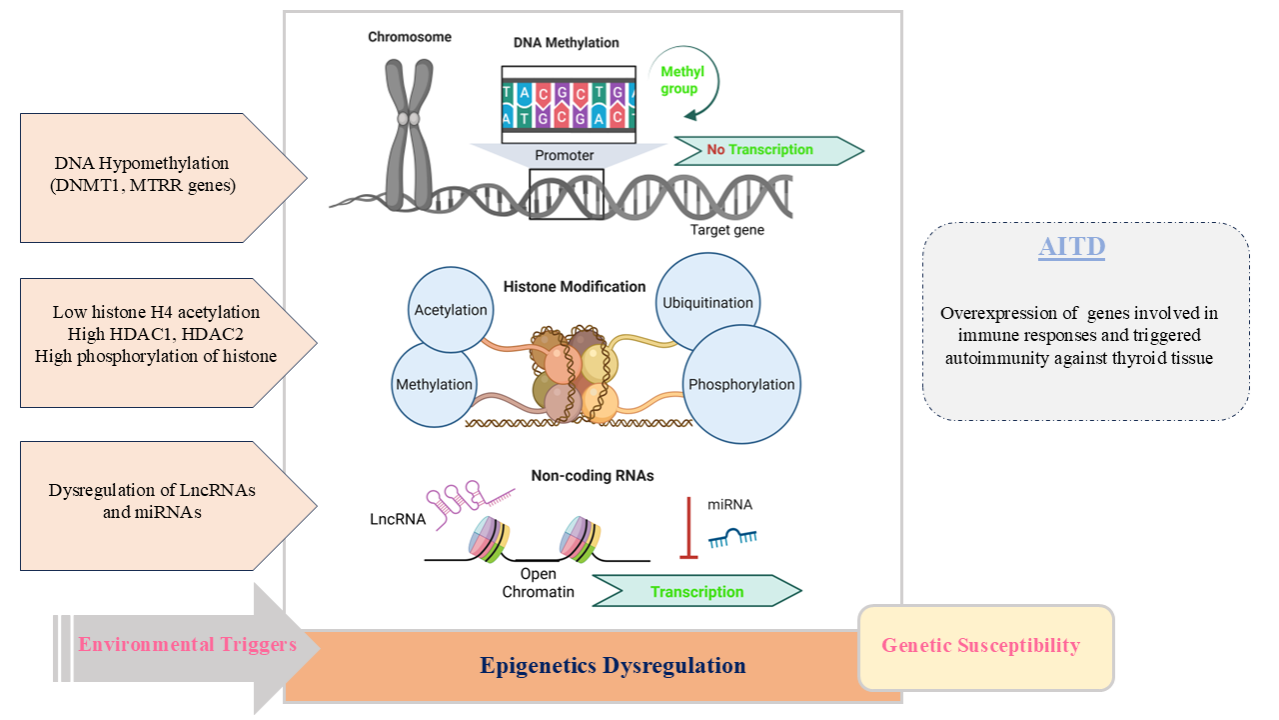
The roles of epigenetic dysregulation in the pathogenesis of AITD. Epigenetic dysregulation plays a crucial role in bridging genetic susceptibility and environmental triggers in AITD. They include DNA methylation, histone modifications, and non-coding RNAs.

Epigenetic modifications, including DNA methylation, histone modifications, and non-coding RNAs, can be regarded as indirect contributors to the coexistence of autoimmune diseases, which play a crucial role in bridging genetic susceptibility and environmental triggers in AITD, influencing immune responses and thyroid-specific gene expression patterns that are fundamental to disease pathogenesis (Feil and Fraga, 2012[37], Tomer, 2014[99], Wang et al., 2017[106]) (Figure 8(Fig. 8)).

Multiple polymorphisms in genes affecting methylation, including DNA methyltransferase 1 (DNMT1) and methionine synthase reductase (MTRR), have been linked to reduced DNA methylation and increased susceptibility to AITD (Arakawa et al., 2012[6]). Moreover, the C677T polymorphism in 5,10-methylenetetrahydrofolate reductase (MTHFR) has been associated with both Graves' disease and Graves' ophthalmopathy (Lee et al., 2016[56]), providing further indirect support for the pivotal role of DNA methylation in AITD development. Histone modification patterns are also altered in AITD. In GD patients, studies have reported decreased histone H4 acetylation, heightened histone deacetylase (HDAC) activity, and dysregulation of active histone marks at immune-related genes (Yan et al., 2015[114]). External factors such as viral infections and cytokines, including interferon-alpha, can modulate thyroid-specific gene expression through their impact on histone remodeling (Lee et al., 2023[55]).

Non-coding RNAs, particularly microRNAs (miRNAs), are significantly dysregulated in AITD. Notably, miR-155-5p and miR-146a-5p are found at reduced levels in both thyroid tissue and immune cells of patients with GD and HT (Bernecker et al., 2012[11]). These miRNAs are involved in regulating immune signaling and inflammation, with an inverse correlation observed between miR-146a-5p and IL-17 levels in Graves' ophthalmopathy (Bernecker et al., 2012[11]).

Previous findings also highlighted the involvement of long non-coding RNAs (lncRNAs) in AITD pathophysiology. For example, lncRNA *Heg* has been associated with immunological markers in GD, while lncRNA *IFNG-AS1* may contribute to enhanced Th1 responses in HT (Christensen et al., 2008[26]). Furthermore, genetic studies have identified susceptibility loci containing lncRNAs linked to GD, underscoring their potential role in disease onset and progression (Christensen et al., 2011[27]).

## Artificial Intelligence in Autoimmune Disease Diagnosis and Management

The complexity of polyautoimmune conditions poses significant diagnostic and management challenges. Patients may present with a constellation of symptoms attributable to different autoimmune processes, and massive amounts of data from clinical exams, laboratory tests, imaging, and genetic profiles may be available (Anaya, 2014[2], Ullah et al., 2025[103]). In recent years, artificial intelligence (AI) has emerged as a promising tool to tackle this complexity by analyzing large datasets to detect patterns, improve diagnosis, and even guide therapy in autoimmune diseases (Chen et al., 2025[24]). AI, particularly machine learning (ML) and deep learning (DP) techniques, can process high-dimensional data far more quickly than humans, identifying subtle correlations that might predict disease or treatment responses (Mane et al., 2024[67]).

One of the most impactful applications of AI in autoimmunity is in early diagnosis and risk prediction. In thyroidology, AI has been applied to interpret thyroid ultrasounds and scans (Cao et al., 2023[20], Li et al., 2023[59]). Notably, the thyroid was one of the earliest organs where AI was tested for diagnosis, as far back as 1991, attempts were made to use neural networks to diagnose thyroid disorders from lab tests (Zhang and Berardi, 1998[119]). Today, deep learning algorithms can analyze thyroid ultrasound images to detect signs of autoimmune thyroiditis, such as hypoechogenicity and heterogeneity of the thyroid texture (Zhang et al., 2022[120]). This is especially useful for detecting seronegative autoimmune thyroiditis called SN-CAT, or seronegative chronic autoimmune thyroiditis, where a patient exhibits clinical features of Hashimoto's but has negative TPO/Tg antibodies (Wu et al., 2025[113]). In fact, combining an experienced sonographer's assessment with AI assistance has been shown to improve diagnostic accuracy for thyroid conditions. 

Beyond diagnosis, AI is being leveraged to enhance the management of autoimmune diseases, including those affecting the thyroid. One straightforward approach is through pattern recognition in large electronic health record datasets to identify which treatments are most effective for specific patient subsets. Machine learning can evaluate among patients with Hashimoto's hypothyroidism who also have another autoimmune disease, what proportion responds well to a certain thyroid hormone dose or needs combination T3/T4 therapy (Zammit and Sykes, 2024[117], Ngan et al., 2025[74]). While this is still an emerging area, it holds promise for personalized medicine.

## Conclusion and Future Perspectives

AITD is a key factor in polyautoimmunity, often occurring alongside other organ-specific autoimmune disorders like type 1 diabetes, Addison's disease, and autoimmune gastritis. This overlap arises not by chance, but due to shared genetic factors, immune system dysfunction, and environmental factors. Various mechanisms, including HLA haplotypes, FOXP3 and CTLA-4 gene variants, Treg/Th17 imbalances, and epitope spreading, contribute to a shared autoimmune foundation. Consequently, AITD frequently acts as an early indicator and a potential entry point for wider autoimmune conditions. Understanding AITD within this broader framework has significant clinical implications. It calls for a proactive and integrated approach to patient care that transcends single-disease management, focusing instead on vigilant screening for coexisting autoimmune disorders. Identifying at-risk individuals through genetic and serological markers, like shared HLA alleles or the presence of multiple autoantibodies, can support early diagnosis and intervention. Furthermore, recognizing patterns such as thyrogastric or autoimmune polyendocrine syndromes allows clinicians to anticipate disease progression and tailor treatment strategies accordingly.

In the future, therapies that emphasize immune tolerance instead of merely individual organ function offer a promising path. Strategies that adjust T-cell activity, enhance regulatory mechanisms, or alter harmful cytokine profiles, such as IL-17, IL-6, and IFN-γ, could potentially address multiple diseases simultaneously. While still in the research phase, antigen-specific tolerogens for the thyroid and other tissues may ultimately lead to preventive or disease-modifying treatments for patients with a genetic predisposition to polyautoimmunity. AI is also poised to play a transformative role in this evolving landscape. From identifying subtle early markers of disease to predicting which patients are at risk of developing multiple autoimmune disorders, AI-driven models offer the potential for earlier diagnosis and more precise risk stratification. Integrating AI with multi-omics data and clinical variables could further refine individualized treatment plans and enable dynamic disease monitoring using wearable technologies.

However, despite this promising outlook, several important limitations must be acknowledged before such therapeutic innovations can be safely and effectively applied in the context of AITD and polyautoimmunity. While immune-tolerance therapies such as antigen-specific tolerogens, tolerogenic dendritic cells (TolDCs), and adoptive Treg transfer hold considerable promise for treating AITD within a broader autoimmune context, their clinical translation faces substantial hurdles (Phillips et al., 2019[78], Nikolic et al., 2020[75], Selck and Dominguez-Villar, 2021[90]). The selection of disease-relevant autoantigens remains problematic, complicating antigen-specific approaches and reducing durability due to epitope spreading and the presence of low-frequency autoreactive T cells. TolDCs have demonstrated safety in early trials (notably in type 1 diabetes), but their efficacy is limited by phenotypic instability in inflammatory environments, variability due to host metabolic state, the need for co-treatments to maintain tolerogenic phenotype, and logistical challenges in ex vivo manufacturing and optimal route of administration (Funda et al., 2019[43], Mansilla et al., 2023[68]). Similarly, antigen-specific Treg therapies require precise antigen targeting and in vivo stability, both of which remain elusive at scale (Selck et al., 2021[90]). Furthermore, cytokine modulation strategies, targeting IL-17, IL-6, and IFN-γ, carry the risk of unintended immunosuppression, infection, or malignancy, and their efficacy across heterogeneous autoimmune phenotypes remains unclear (Siwakoti et al., 2023[94]). Finally, AI-assisted predictive and therapeutic tools, although transformative in concept, face limitations in model validation, data integration, computational scalability, transparency, and bias, issues that undermine generalizability across diverse populations unless rigorously addressed (Mane et al., 2024[67], Liu et al., 2025[61]).

Altogether, while AITD is not just a solitary thyroid issue but often a manifestation of broader autoimmune dysregulation, future treatment strategies must carefully balance innovation with feasibility, safety, and precision. By acknowledging these limitations, healthcare systems can more effectively manage patients with multiple autoimmune diseases, mitigate long-term risks, and move closer to the goal of comprehensive, preventive autoimmune care.

## Declaration

### Ethics approval and consent to participate

Not applicable.

### Consent for publication

Not applicable.

### Availability of data and materials

Not applicable.

### Competing interest

The authors declare that they have no competing interest.

### Artificial Intelligence (AI) - Assisted Technology

The authors declare that AI was not used for the preparation of this manuscript.

### Funding

The authors declare that they have no funding support.

### Authors' Contribution

ShH, RA, and MH designed and drafted the manuscript, collected the references, and carried out the primary literature search. ShH, RA, MZ and MH modified the manuscript and participated in discussions. All authors read and approved the final manuscript.

## Figures and Tables

**Table 1:**
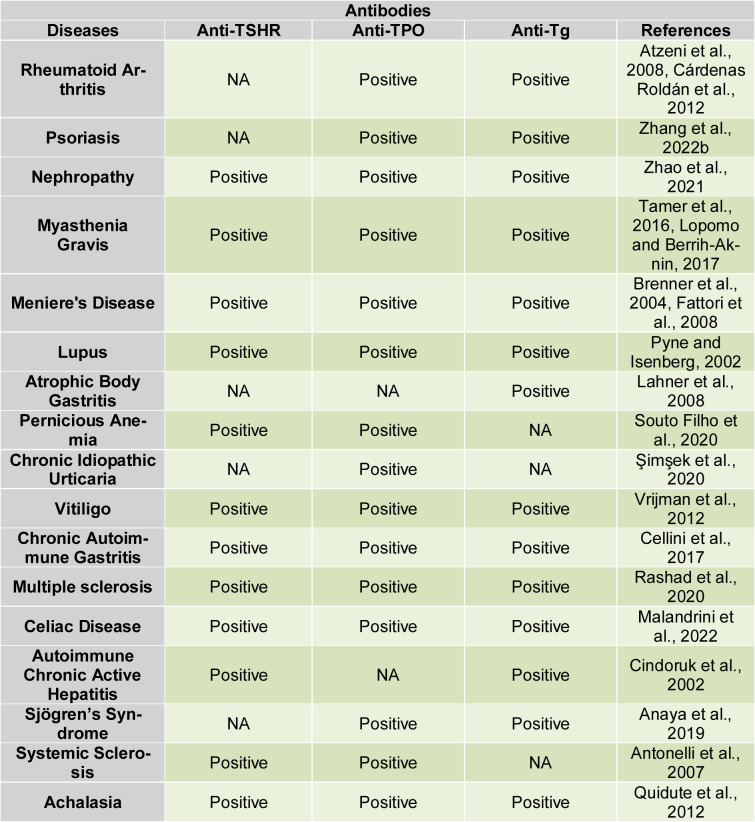
The prevalence of thyroidal autoantibodies in other autoimmune diseases

**Figure 1:**
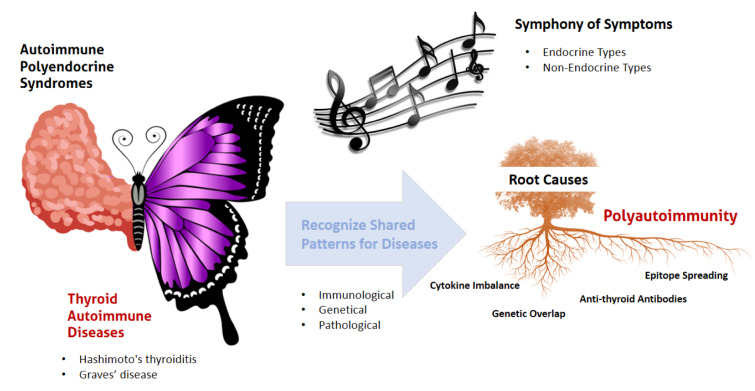
Graphical abstract
